# Association between cervical spine clinical active range of motion and pain or disability in people with neuromusculoskeletal neck pain: A systematic review and meta-analysis

**DOI:** 10.1371/journal.pone.0353504

**Published:** 2026-07-24

**Authors:** Saghar Soltanabadi, Sima Vatandoost, Mohammad Bayattork, Michael J. Lukacs, Alison Rushton, David M. Walton

**Affiliations:** 1 School of Physical Therapy, Western University, London, Ontario, Canada; 2 Department of Sport Sciences and Physical Education, Faculty of Humanities Science, University of Hormozgan, Bandar Abbas, Iran; 3 Physiotherapy Department, London Health Science Center (LHSC), London, Ontario, Canada; Mazandaran University of Medical Sciences, IRAN, ISLAMIC REPUBLIC OF

## Abstract

**Objectives:**

To quantify the association between clinical cervical active range of motion and patient-reported outcomes of pain and disability in people with neuromusculoskeletal neck pain.

**Design:**

Systematic review and meta-analysis following Preferred Reporting Items for Systematic Reviews and Meta-Analyses guidelines, registered with PROSPERO (CRD42023417317).

**Methods:**

A comprehensive search of six databases and grey literature up to December 2024 identified observational studies reporting cross-sectional correlations between active range of motion and patient-reported outcomes in adults with neck pain. Risk of bias was assessed using an adapted National Institutes of Health quality assessment tool. Eight meta-analyses were conducted using random-effects models. Heterogeneity, subgroup analyses (acute versus chronic neck pain, whiplash-related versus non-traumatic), and meta-regressions (age, sex, publication year, risk of bias) were performed. Certainty of evidence was assessed using the Grading of Recommendations Assessment, Development and Evaluation approach.

**Results:**

45 studies (total N = 3,494) were included. Meta-analyses showed statistically significant small to moderate negative correlations (r = −0.21 to −0.34) between active range of motion and patient-reported outcomes in all planes of motion, with stronger associations in acute and whiplash-related neck pain. Substantial heterogeneity (I^2^ = 67–91%) was partly explained by symptom duration and mechanism of onset. Certainty of evidence was very low due to risk of bias, inconsistency, and imprecision.

**Conclusions:**

Cervical active range of motion is modestly associated with pain and disability, especially in acute and traumatic neck pain. Findings should be interpreted with caution. Standardized measurement protocols and higher-quality studies are needed to inform practice.

## Introduction

Neck pain (NP) is a prevalent condition with a point prevalence of 7.6% and a lifetime prevalence of nearly 50% globally [1]. Beyond physical discomfort, NP is a leading contributor to disability [2], psychological distress [3,4], and economic burden due to healthcare costs and lost productivity [5]. Despite ongoing research, the prevalence of NP and its associated disability have remained largely unchanged over the past three decades [6] highlighting the need for more effective diagnostic and therapeutic strategies.

Active range of motion (AROM) of the cervical spine is one of the most commonly assessed physical impairments in clinical practice [7], and is considered important for evaluating pain and functional limitations in patients with mechanical neck disorders [8,9]. The 2017 APTA NP Clinical Practice Guidelines recommends using AROM, alongside other clinical findings, to classify patients into four subtypes (mobility deficits; movement coordination impairments, including whiplash‑associated disorders (WAD); cervicogenic headaches; and radiating pain) and to guide tailored intervention strategies [10]. Prior systematic reviews have demonstrated group mean differences in AROM between people with and without NP [11,12], supporting the construct validity of AROM as an impairment‑level measure in this population. However, these reviews did not systematically synthesize the strength and consistency of associations between cervical AROM and patient‑reported outcomes (PROs) such as pain intensity and disability, which are central to clinical decision-making. Clinical Practice Guidelines for NP consistently endorse assessment and treatment of neck mobility [13–15], through often implicit assumptions that pain reduces mobility and improving mobility will improve pain (or vice versa). If AROM impairments are strongly correlated with PROs, this would support clinical guidelines and suggest mobility metrics could serve as a practical observational clinical proxy for subjective symptom severity. Conversely, if the correlation is weak, reliance on AROM alone may lead to incomplete or misleading evaluations or mistargeted treatment strategies.

Despite the frequent use of AROM to monitor rehabilitation progress [7,16,17] and guide exercise prescription [18,19], the magnitude and consistency of its association with Patient‑Reported Outcome Measures (PROMs) remains unclear [8,20]. Individual studies have reported inconsistent associations between cervical AROM and PROMs. For example, Vernon et al. reported a moderate negative association between total cervical AROM and disability (r = −0.58) [21], whereas Missman et al, reported little to no association between comparable measures of total AROM and disability (r ≈ 0.00) [22]. This variability in effect estimates across studies suggests that the relationship between cervical AROM may depend on factors such as NP subgroup, symptom duration, or differences in measurement protocols and outcome instruments. The presence of these discordant results in the primary literature highlights a critical knowledge gap especially in light of the burden of neck pain and the frequency with which people with neck pain seek rehabilitation therapies [23]. Therefore, a systematic review and meta-analysis is needed to address this gap and to synthesize the available evidence, estimate the overall magnitude of association, and explore potential sources of heterogeneity.

### Objective

To systematically search, appraise, and quantitatively synthesize literature on the magnitude of association (correlation) between clinical AROM and PROs of cervical pain or disability in adults with neuromusculoskeletal (non-cancer, non-infection, non-fracture) NP.

## Methods and analysis

### Design

Following the Preferred Reporting Items for Systematic Review and Meta-Analyses (PRISMA), this review is part of a planned series of reviews that will explore the correlation between clinical biomechanical metrics of cervical spine (AROM, strength, proprioception, etc.) and pain or disability in people with NP. The protocol [24] is registered with PROSPERO (CRD42023417317) and has been published [24]. The completed PRISMA 2020 checklist is provided as S4 File.

### Deviation from protocol

Although the registered protocol stated that non-English studies would be excluded, advances in translation tools made inclusion accessible. Eligible non-English full-texts were translated using AI-assisted translation (ChatGPT 5) and verified by a bilingual reviewer for accuracy.

### Eligibility criteria

Observational studies were included [24,25] if they reported and evaluated a cross-sectional association between at least one plane of clinical AROM and at least one standardized and validated PROM [26–28] of NP or disability in adults with neuromusculoskeletal conditions. Studies including more than 50% of participants diagnosed with “Temporomandibular Disorders”, “Vertigo” and “Dizziness” were excluded during screening to reduce heterogeneity.

To ensure the findings are relevant within the context of rehabilitation, “clinical AROM” was defined as quantification of volitional maximal movement of the neck [29] that can be feasibly observed or captured by a third-person evaluator in a rehabilitation clinical environment. These measures are immediately meaningful to clinicians and do not require high-priced equipment (e.g., imaging) or any further computation, ensuring their relevance and utility in clinical settings. Data on AROM were sought for all planes of movement: coronal (lateral flexion), sagittal (flexion/extension), and horizontal (rotation) to the right and left where relevant.

### Information sources

A systematic search was conducted in MEDLINE, Embase, Scopus, Web of Science, SPORTDiscus, and CINAHL from inception to December 8, 2024. Grey literature, including conference abstracts/proceedings and dissertations, were also sought from Embase, Scopus, ProQuest, and Grey Matters. The reference lists of included articles and relevant reviews were hand-searched for additional studies. The search strategy, initially developed in MEDLINE Ovid (S1 File), was tailored for the other databases [24].

### Data management and selection

**Fig 1:**
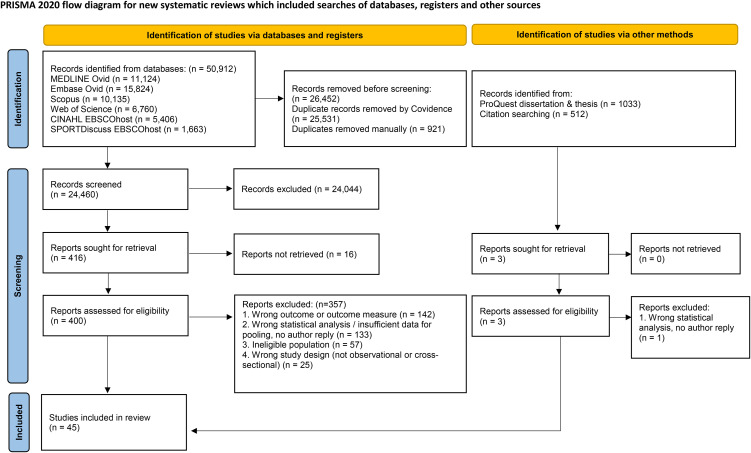
PRISMA Flow Diagram.

Literature search results were imported into Covidence [30,31] where duplicates were removed. Two reviewers screened titles and abstracts (SS, SV) and full-text studies (SS, MB). Disagreements were resolved through consensus. Perfect agreement was achieved in both stages of selection process after discussion and reasons for exclusion at full-text stage were documented in the PRISMA flow diagram (Fig 1) [25].

### Data collection process and Items

Study data were extracted independently by two reviewers (SS, MB), using a custom data extraction form. Extracted data included: study characteristics, participant demographics, definitions/classifications of NP, and key outcomes (e.g., pain, disability, or both, and how measured). Study level information was later used in meta-analysis to explain the heterogeneity via meta-regression techniques. If data were incomplete or unreported, and the corresponding author could not be reached after two emails over two weeks, only available data were extracted.

Pain and disability were treated as distinct outcomes, and if a study reported both, they were extracted and pooled separately in the meta-analyses. In studies where multiple (Patient-reported Outcome Measures) PROMs of the same construct (e.g., disability) were reported in the same cross-sectional time frame (e.g., Neck Disability Index (NDI) [32] and Northwick Park Neck Pain Questionnaire (NPQ) [33]), one outcome was selected for inclusion in analyses and reporting. The selection was based on the outcome that provided the most complete information for analysis. If no PROMs were excluded, the outcome that was most reported across selected studies when pooled in a meta-analysis was chosen, typically the NDI as the most commonly used neck-specific disability PROM [32]. As a final step to optimize analytic power for the disability construct, recognizing the strong correlation between NPQ and NDI of r = 0.88 [34], we pooled data from studies that used either tool (one tool per study) and evaluated for heterogeneity before presenting this as a pooled ‘AROM v Disability’ analysis.

### Risk of bias assessment

Risk of bias (RoB) was assessed using an adapted version of the National Institute of Health (NIH) Quality Assessment Tool for Observational, Cohort, and Cross-Sectional Studies (QATOCCS) [35], due to its versatility across observational study designs (i.e., cross-sectional, cohort and case-control) and previous use in similar reviews [36–38]. The adaptations are detailed in S2 File. Two reviewers (SS, MB) independently assessed each included study, with discrepancies resolved through discussion. Studies were classified based on “Yes” responses to the assessment criteria: > 80% as “Low,” 60–80% as “Moderate,” and <60% as “High” RoB [39]. RoB assessments primarily relied on published data to standardize the interpretation.

### Data synthesis

The “metafor” package [40] in R [41], and RStudio add-on [42] was employed to conduct meta-analyses and generate all plots [43]. Due to differing characteristics of NP populations, such as age, sex, and chronicity of pain, the random-effects model was employed to account for the diversity of effect sizes across studies while not being overly influenced by any single study [44].

Per Cochrane recommendations [45], each pooled meta-analysis was only performed when ≥3 primary studies were available to provide a minimally stable estimate of between-study heterogeneity and effect size; meta-regression analyses were limited to outcomes with ≥10 studies; and subgroup comparisons required at least two studies per subgroup to ensure interpretability.

### Choice of effect measure

The primary effect measure used was Pearson’s r and Spearman’s ρ, which quantify the strength and direction of the linear relationship between two continuous variables of AROM (degrees) and pain/disability outcomes (converted to percentage scores to standardize scaling for presentation).

### Transformation and synthesis

Initially, all correlation coefficients were converted to Fisher’s *Z* values for the purpose of meta-analyses to stabilize variances and harmonize effect metrics [46]. Plane-level *Z* values (sagittal, coronal, transverse) were then pooled via inverse-variance–weighted averaging in R, yielding one *Z* per plane. In studies lacking a total AROM (a composite index of degrees of motion across all three cardinal planes of cervical motion) correlation but reporting all three plane-level correlation coefficients, those values were similarly combined to derive an overall cervical AROM correlation with pain or disability. Final pooled *Z* estimates were back-transformed to Pearson’s *r* for interpretability.

### Interpretation and comparison of effect sizes

Strength of the correlation (regardless of direction) were interpreted as small (0.10 ≤ r ≤ 0.29), medium (0.30 ≤ r ≤ 0.49), and large (≥ 0.50) [47,48]. To determine whether cervical AROM was more strongly associated with disability than with pain, paired comparisons of pooled associations were performed for each plane of motion. For each comparison, the difference between the estimates ($Z_{disability}$ and $Z_{pain}$) was divided by the square root of the sum of their squared standard errors ($\sqrt{{SE}_{disability}^{\text{2}}+{SE}_{pain}^{\text{2}}}$) to yield a standard normal test statistic $Z_{diff}$. Two‑tailed p values were obtained from the standard normal distribution, and significance was set at p < 0.05. Additionally, to assess variation across planes, the same Z‑test was applied within each outcome domain, comparing every pair of pooled Fisher’s Z values (e.g., Sagittal AROM vs. Coronal AROM) for both disability and pain.

### Assessment of heterogeneity, meta-regression and subgroup analysis

Heterogeneity across studies was evaluated using the Cochran’s Q Test and I^2^ statistic as proportion of total variation across studies and τ² as an estimate of between-study variance, that is due to heterogeneity rather than chance. I² weas interpreted according to the guidelines of the Cochrane handbook [49]. To explore potential sources of heterogeneity, four meta-regressions of study-level variables were planned *a priori* based on their relevance to the study populations and potential influence on the outcomes of interest [24]: 1) Participants’ mean age (as continuous variable), 2) Percentage of female sex (continuous), 3) RoB of included studies (continuous variable between 0–11), 4) Article publication year (as continuous variable). Two additional dichotomous subgroup analyses were also planned: 1) Duration of pain: acute/subacute (≤ 8 weeks) vs. chronic (> 8 weeks). 2) Mechanism of onset: traumatic (i.e., whiplash) vs. non-traumatic. Whiplash status was inferred from study objectives and inclusion criteria, allowing categorization by presence or absence of whiplash but not by specific WAD grades [50].

Studies were always retained in the plane-level meta-analyses but were omitted from any meta-regression or subgroup analysis for which requisite study-level information (e.g., pain duration or whiplash status) was not reported.

### Sensitivity analyses

A leave-one-out sensitivity analysis was conducted to assess the robustness of the synthesized results and identify any influential studies that may disproportionately affect the meta-analysis outcomes.

### Certainty assessment

The Grading of Recommendations Assessment, Development, and Evaluation (GRADE) [51] approach was used by two reviewers independently (SS, MB) to evaluate the certainty of evidence (association) for each meta-analysis outcome. Discrepancies in ratings were resolved through team discussions, ensuring the reliability and consistency of the final judgments.

According to GRADE guidelines for observational studies [52–54], the evidence was initially assigned a low certainty rating to each outcome (correlation) which could be further downgraded or upgraded based on several factors. Evidence was downgraded if over 25% of participants were from studies with a high RoB [55,56]. Inconsistency in the evidence, indicated by substantial I^2^ (50% to 90%) or considerable I^2^ (75% to 100%) without clear explanation, warranted a downgrade [49,57]. Indirectness was assessed by how closely studies aligned with the population of interest, with deviations leading to potential downgrading [58]. Imprecision was evaluated based on the width of confidence intervals, with wide intervals or non-significant effect sizes resulting in a lower certainty rating [59]. Potential publication bias was assessed using funnel plots, Egger’s test, and rank correlation tests [60]. Conversely, evidence could be upgraded for large effect sizes (r ≥ 0.50) [61,62].

## Results

The searches identified 50,912 database records and 1,545 additional records. After duplicate removal and screening, 403 reports were assessed for eligibility. Ultimately, 45 studies examining associations between cervical spine AROM and PROMs of pain or disability were included (Fig. 1). Forty-two studies were peer-reviewed journal articles [21,22,63–102], along with one PhD thesis [103] and two conference abstracts [104,105]. Additional data were provided by authors for 13 studies [22,84–86]. Reasons for exclusion are reported in the S3 File.

### Study characteristics

The 45 included studies encompassed 3,494 participants (sample sizes: 12–599) published from 1997–2024. NP conditions varied and included whiplash, idiopathic pain, cervicogenic headache, cervical spondylosis, discopathy, and non-specific NP (S1 Table). Pain was assessed in 36 studies (Visual Analog Scale: in 21 [64,65,67,68,71–73,77–79,82,84,85,87–89,99,101,103–105]; Numeric Pain Rating Scale: in 11 [22,66,75,76,80,83,92,93,95–97]; Verbal Numeric Pain Scale: in 4 studies [21,69,74,81]). Disability was reported in 41 studies, primarily using the NDI (in 38 [21,22,63,64,66–68,70–72,74–76,78–82,86–101,103–106]) or NPQ (in 3 studies [69,83,84]). AROM was primarily measured using goniometers such as Cervical Range of Motion device (CROM) (in 30 [21,22,63,64,66–68,70,72,73,75,78–82,86–89,93,95–98,100,103–106]), wearable inertial sensors (in 4 [76,91,94,99]), digital inclinometers (in 4 studies [65,74,90,92]), and other tools (S1 Table).

### Risk of bias

RoB scores ranged from 2 to 9 out of 11 (mean: 5.3). Only one study was rated low RoB, nine moderate, and 35 high (S2 Table).

### Results of syntheses and certainty of evidence

Eight meta-analyses evaluated associations between cervical AROM and pain/disability, grouped by anatomical planes (total, sagittal, transverse, coronal). Meta-regression and subgroup analyses explored heterogeneity by sex (percentage female), publication year, chronicity (acute/subacute vs. chronic), and mechanism of onset (whiplash vs. non-whiplash). The pooled correlations, heterogeneity statistics, significant moderators, subgroup analyses, corresponding coefficients of determination, and leave-one-out analysis ranges are presented comprehensively in Table 1. Certainty of evidence was very low for all eight pooled correlations as shown in Table 2 (Full details in S3 Table).

**Table 1:** Meta-analyses Results.

AROM^a^ Plane	Studies (N^b^)	EE^c^ [95% CI]	Model P	Q-test P	I² (%)	R² (%)	LOO^d^ (r range)
**Disability**							
**Total**	23 (2011)	r^e^: −0.30 [−0.39, −0.20]	< 0.001	< 0.001	75.6	—	−0.29 to −0.33
MR^f^: Female%^g^	23	S^h^: 0.004 [0.001, 0.006]	0.009	< 0.001	69.4	25.8	—
MR: Pub-Year^i^	23	S: 0.02 [0.004, 0.03]	0.007	< 0.001	68.1	29.7	—
Chronicity^j^	15	D^k^: 0.40 [0.10, 0.60]	0.002	0.02	52.7	57.2	—
Whiplash^l^	21	D: −0.30 [−0.50, −0.10]	0.003	< 0.001	63.6	39.8	—
**Sagittal**	27 (1818)	r: −0.28 [−0.42, −0.13]	< 0.001	< 0.001	90.9	—	−0.27 to −0.36
Outlier removed	26	r: −0.34 [−0.43, −0.25]	<0.001	< 0.001	71	—	—
MR: Female%	26	S: 0.003 [0.0004, 0.006]	0.03	< 0.001	65	19.8	—
Chronicity	21	D: 0.25 [−0.02, 0.51]	0.07	< 0.001	64.1	11.5	—
Whiplash	23	D: −0.23 [−0.47, 0.02]	0.07	< 0.001	65.2	10.9	—
**Transv.** ^m^	24	r: −0.29 [−0.37, −0.20]	<0.001	< 0.001	67.7	—	−0.28 to −0.31
MR: Female%	24	S: 0.003 [0.0002, 0.006]	0.04	< 0.001	63.1	15.4	—
Chronicity	19	D: 0.29 [0.06, 0.53]	0.01	0.003	57.6	35.4	—
Whiplash	22	D: −0.28 [−0.47, −0.09]	0.004	0.005	50.2	44.9	—
**Coronal**	17 (1411)	r: −0.28 [−0.39, −0.16]	<0.001	< 0.001	76.6	—	−0.26 to −0.32
MR: Female%	16	S: 0.004 [0.001, 0.007]	0.01	< 0.001	66.7	39.5	—
MR: Pub-Year	17	S: 0.02 [0.004, 0.04]	0.01	< 0.001	67.1	30.9	—
Chronicity	13	D: 0.37 [0.04, 0.69]	0.03	< 0.001	70.4	29.8	—
Whiplash	15	D: −0.37 [−0.63, −0.10]	0.007	< 0.001	65.2	39.4	—
**Pain**							
**Total**	15 (1,888)	r: −0.26 [−0.36, −0.16]	<0.001	< 0.001	75	—	−0.23 to −0.28
Chronicity	10	D: 0.28 [0.05,0.5]	0.01	0.006	64.7	48.2	—
Whiplash	15	D: −0.27 [−0.44, −0.11]	< 0.001	0.01	52.1	59	—
**Sagittal**	13 (848)	r: −0.24 [−0.36, −0.12]	< 0.001	< 0.001	70.5	—	−0.29 to −0.21
Chronicity	10	D: 0.40 [0.04, 0.8]	0.03	0.017	58.6	26	—
Whiplash	12	D: −0.35 [−0.8, 0.05]	0.09	< 0.001	69.1	15.6	—
**Transv.**	10	r: −0.27 [−0.41, −0.11]	0.001	< 0.001	74	—	−0.33 to −0.23
Chronicity	7	D: 0.28 [−0.16, 0.71]	0.2	0.002	68.7	14.4	—
Whiplash	10	D: −0.21 [−0.63, 0.21]	0.3	< 0.001	72.5	3.4	—
**Coronal**	9	r: −0.22 [−0.37, −0.06]	0.007	< 0.001	69.2	—	−0.27 to −0.18
Chronicity	7	D: 0.26 [−0.21, 0.72]	0.3	0.005	66.3	1.8	—
Whiplash	9	D: −0.24 [−0.70, 0.22]	0.3	0.002	67	2.5	—

^a^Active Range of Motion (AROM).

^b^Number of participants included in the meta-analysis for that row.

^c^Effect estimate (EE) with 95% confidence interval; for main analyses, EE is the pooled Pearson’s r unless otherwise indicated.

^d^Leave-one-out (LOO) sensitivity analysis; range of r values after sequential removal of individual studies.

^e^Pooled Pearson’s r Correlation Coefficient from meta-analysis.

^f^Meta-Regression (MR).

^g^Proportion of female participants (Female%) in the sample (continuous moderator in meta-regression).

^h^Slope (S) in meta-regression analysis; represents change in correlation coefficient per unit increase in the moderator variable.

^i^Publication year of the studies (continuous moderator in meta-regression).

^j^Subgroup analysis by chronicity (duration) of pain (acute/subacute vs. chronic).

^k^Subgroup difference (D); represents the difference in correlation coefficients between subgroups, with positive values indicating stronger correlations in the comparator subgroup relative to the reference group.

^l^Subgroup analysis by mechanism of injury (whiplash-associated vs. other causes of neck pain).

^m^Transverse.

**Table 2:** GRADE Evidence Profile.

Outcome	RoB^a^	Inconsistency	Indirectness	Imprecision	Pub. Bias ^b^	Overall certainty
Disability & Total	Serious	NS ^c^	NS	NS	ND ^d^	Very low
Disability & Sagittal	Serious	NS	NS	NS	ND	Very low
Disability & Transv.^e^	Serious	NS	NS	NS	ND	Very low
Disability & Coronal	Serious	NS	NS	NS	ND	Very low
Pain & Total	Serious	NS	NS	NS	ND	Very low
Pain & Sagittal	Serious	NS	NS	NS	ND	Very low
Pain & Transv.	Serious	Serious ^f^	NS	NS	ND	Very low
Pain & Coronal	Serious	Serious ^f^	NS	NS	ND	Very low

^a^Risk of Bias (RoB) as the indicator of Limitations In Design: downgraded if >25% of the participants were from studies with a high RoB.

^b^Publication bias (Pub. Bias).

^c^Not Serious (NS).

^d^None Detected (ND); Egger’s and rank-correlation tests were statistically insignificant.

^e^Transverse (Transv.).

^f^Some inconsistency remained after leaving an influential study out (Saavedra Hernandez 2012), and meta-regression results were influenced by a single study (Thoomes 2023) in both meta-analyses of Pain × Transverse and Pain × Coronal.

### Correlation between disability and AROM

a) Total AROM

**Fig 2:**
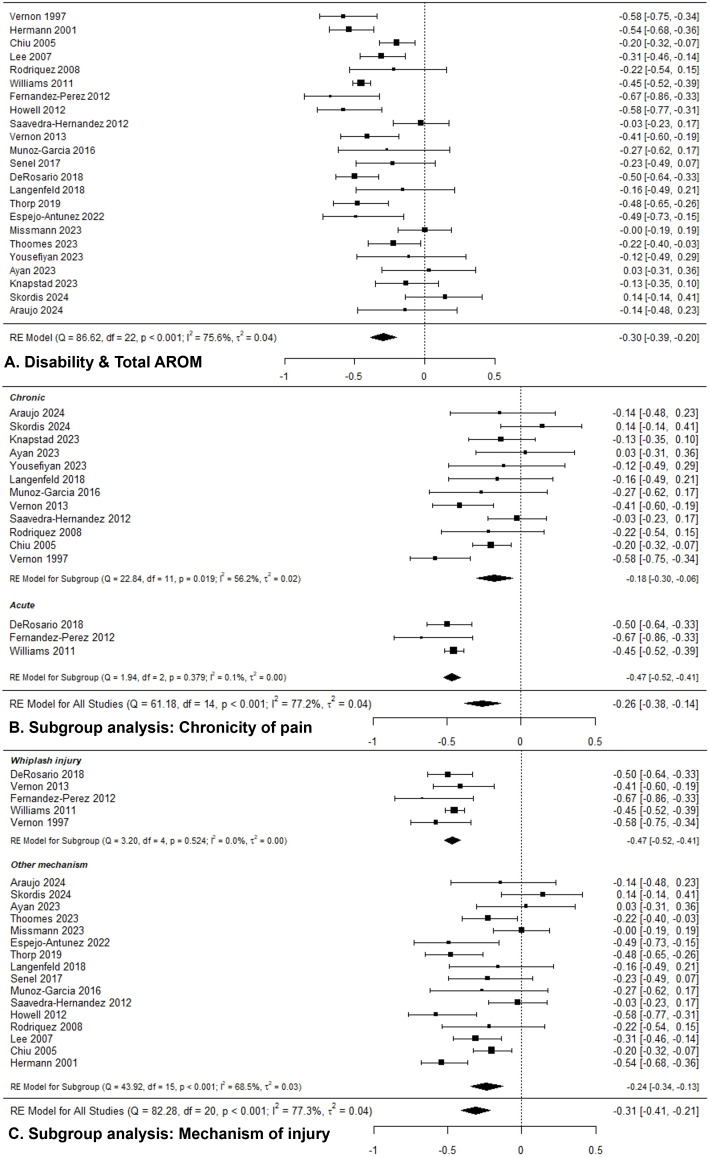
Overall and Subgroup Forest plots: Disability and Total AROM. (A) Overall random-effects meta-analysis (pooled Pearson’s r). (B) Subgroup analysis by chronicity of pain. (C) Subgroup analysis by mechanism of injury. Squares = study estimates (size ∝ inverse-variance weight); horizontal bars = 95% CI; diamonds = pooled effects. Analyses were fit on Fisher’s z and back-transformed to r. Dotted vertical line indicates r = 0. Reported Q, I², and τ² are panel-specific.

**Fig 3:**
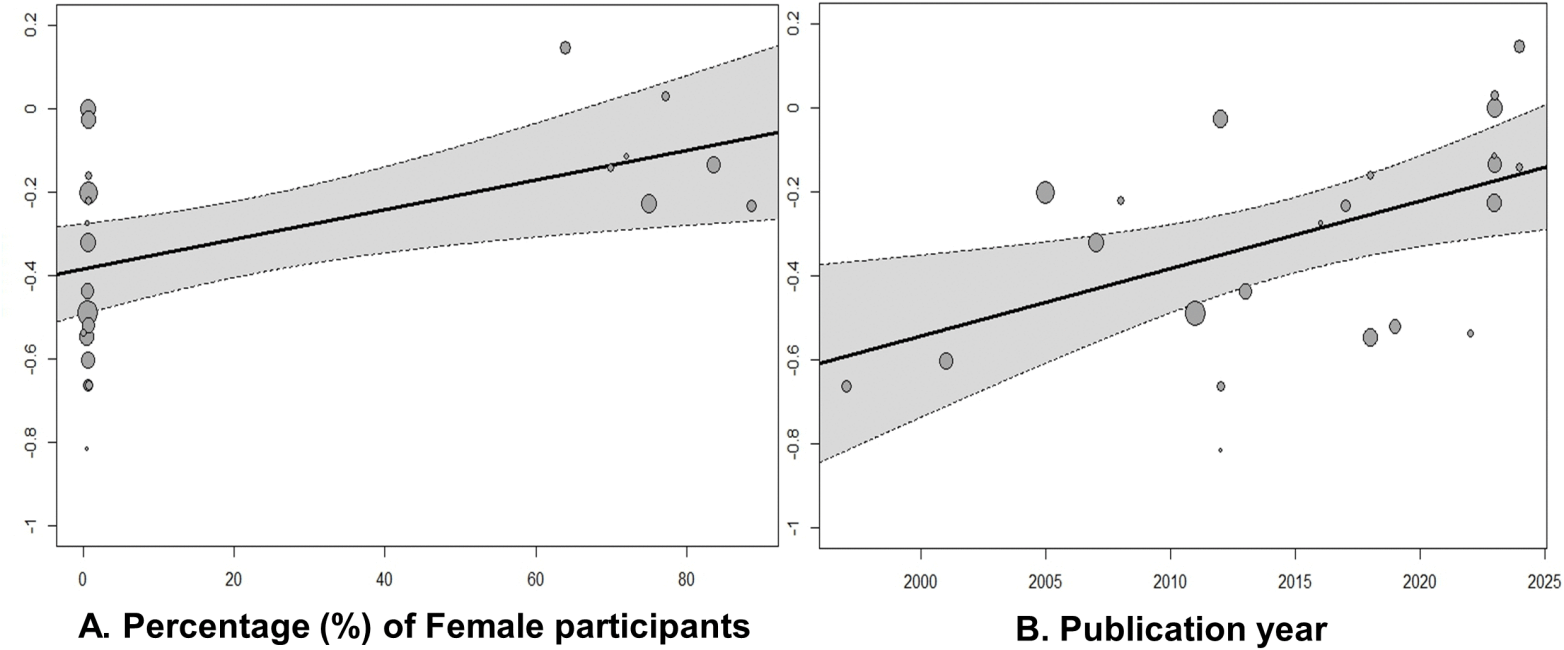
Meta-Regression Bubble Plots: Moderators vs Correlation (Disability & Total AROM). (A) Percentage of female participants vs Fisher’s z; (B) Publication year vs Fisher’s z.

Total AROM (23 studies, N = 2011) showed a significant medium negative association (r = −0.30, 95%CI −0.39 to −0.20; Fig 2A) with substantial heterogeneity (I² = 75.6%). Meta-regression indicated weaker associations with increased female proportions (Fig 3A; p < 0.01; R^2^ = 25.8) and recent publication dates (Fig 3B; p < 0.01; R² = 29.7%). Subgroup analyses showed stronger association in acute/subacute (Fig 2B; p = 0.002; R² = 57.2%) and WAD groups (Fig 2C; p = 0.003; R² = 39.8%). Publication bias was not detected Funnel plot, S1 Fig; Egger’s test, p = 0.3; Kendall’s τ, p = 0.6).

In all Bubble plots, each circle is a study (size ∝ inverse-variance weight). Solid line is the fitted random-effects meta-regression; shaded area represents 95% CI; dashed lines is the prediction interval.

b) Sagittal Plane AROM

**Fig 4:**
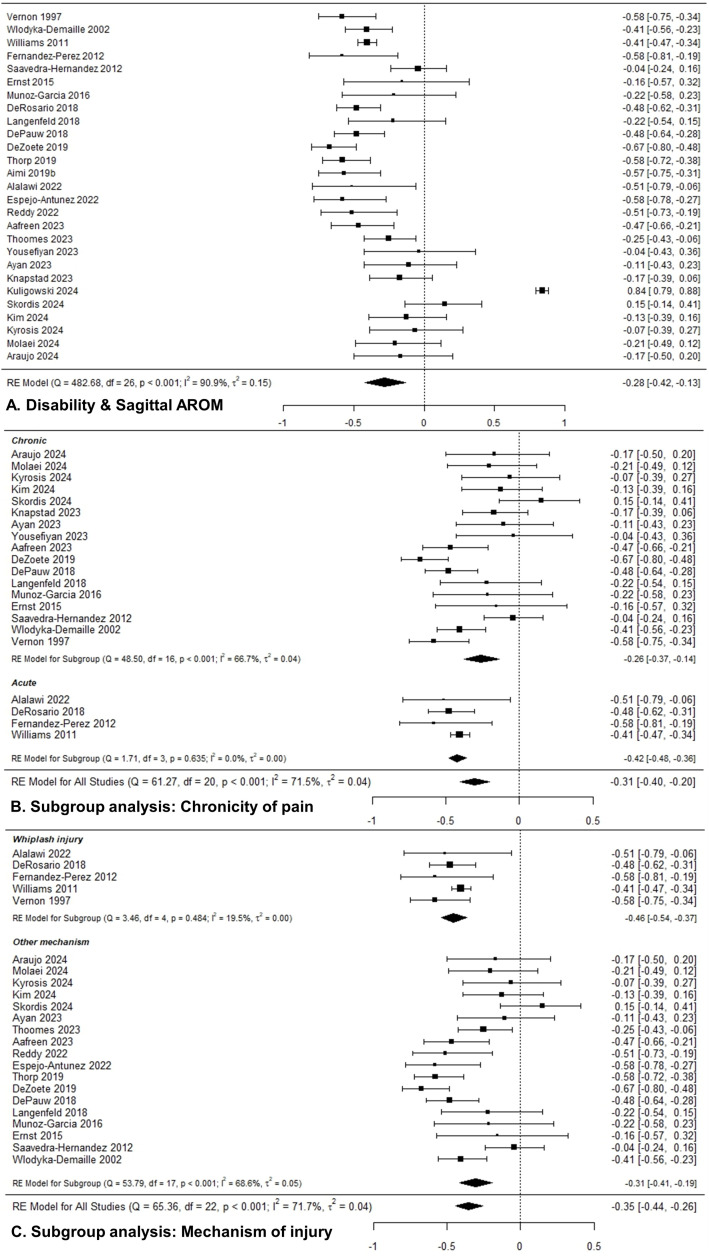
Overall and Subgroup Forest plots: Disability and Sagittal AROM. (A) Overall random-effects meta-analysis before removing outlier (pooled Pearson’s r). (B) Subgroup analysis by chronicity of pain. (C) Subgroup analysis by mechanism of injury.

**Fig 5:**
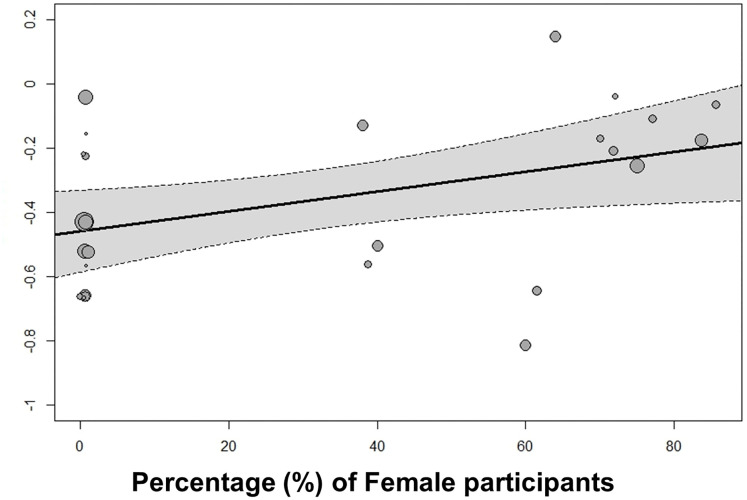
Meta-Regression Bubble Plot: Moderator vs Correlation (Disability & Sagittal AROM). Percentage of Female participants vs Fisher’s z.

Sagittal AROM (26 studies, N = 1639) revealed a significant medium negative correlation (r = −0.34; 95%CI: −0.43 to −0.25) after removing an influential outlier (Kuligowski 2024 [90], r=+0.84), though still showing substantial heterogeneity (I² = 71.0%). Fig 4A shows the Forest plot before removing the outlier. After removing the outlier, meta-regression showed weaker associations with increased female proportions (Fig 5, p = 0.03, R² = 19.8%). Subgroup analyses showed stronger associations for acute/subacute and whiplash groups (Figs 4B and 4C; R^2^ ≤ 11.6%), though not statistically significant (p ≥ 0.06). No publication bias was found (Funnel plot, S2 Fig; Egger’s test, p = 0.5; Kendall’s τ, p = 0.9).

c) Transverse Plane AROM

**Fig 6:**
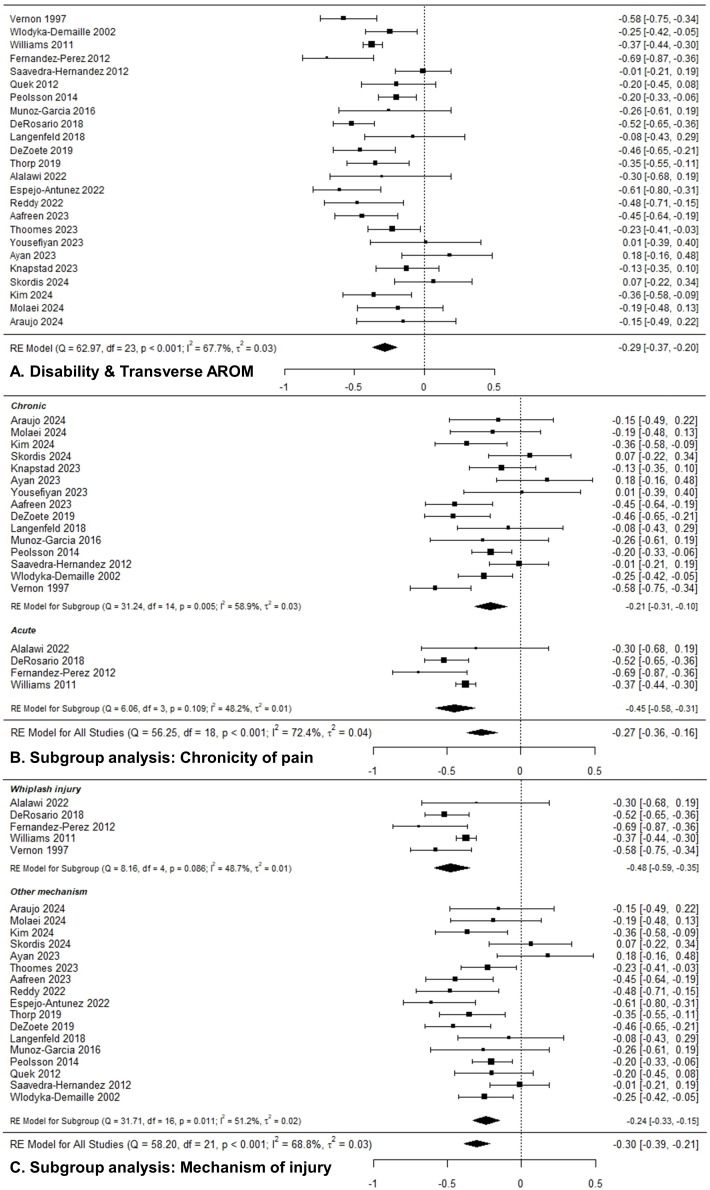
Overall and Subgroup Forest plots: Disability and Transverse AROM. (A) Overall random-effects meta-analysis (pooled Pearson’s r). (B) Subgroup analysis by chronicity of pain. (C) Subgroup analysis by mechanism of injury.

**Fig 7:**
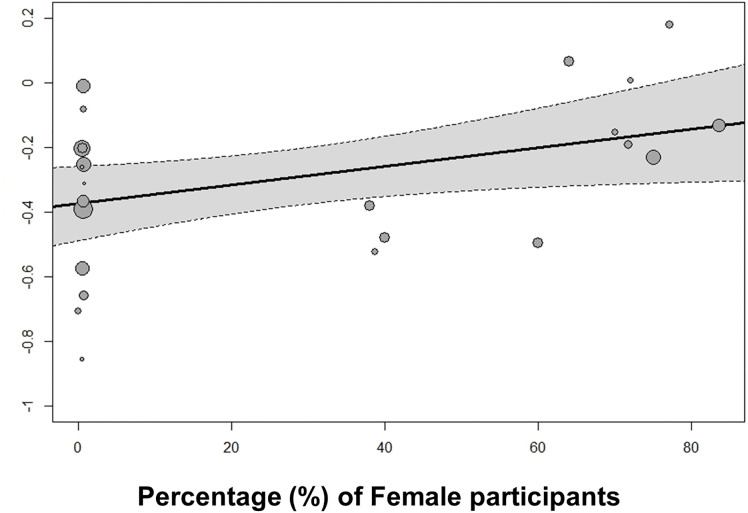
Meta-Regression Bubble Plot: Moderator vs Correlation (Disability & Transverse AROM). Percentage of Female participants vs Fisher’s z.

Transverse AROM (24 studies, N = 1901) demonstrated a significant small-to-medium negative correlation (r = −0.29; 95%CI: −0.37 to −0.20; Fig 6A). Heterogeneity was substantial (I² = 67.7%). Meta-regression showed weaker associations with increased female proportions (Fig 7, p = 0.03, R² = 15.4%). Subgroup analyses confirmed stronger associations for acute/subacute (Fig 6B, p = 0.01; R^2^ = 35.40%) and WAD cases (Fig 6C, p = 0.004; R^2^ = 44.9%). No publication bias was detected (Funnel plot, S3 Fig; Egger’s test, p = 0.56; Kendall’s τ, p = 0.60).

d) Coronal Plane AROM

**Fig 8:**
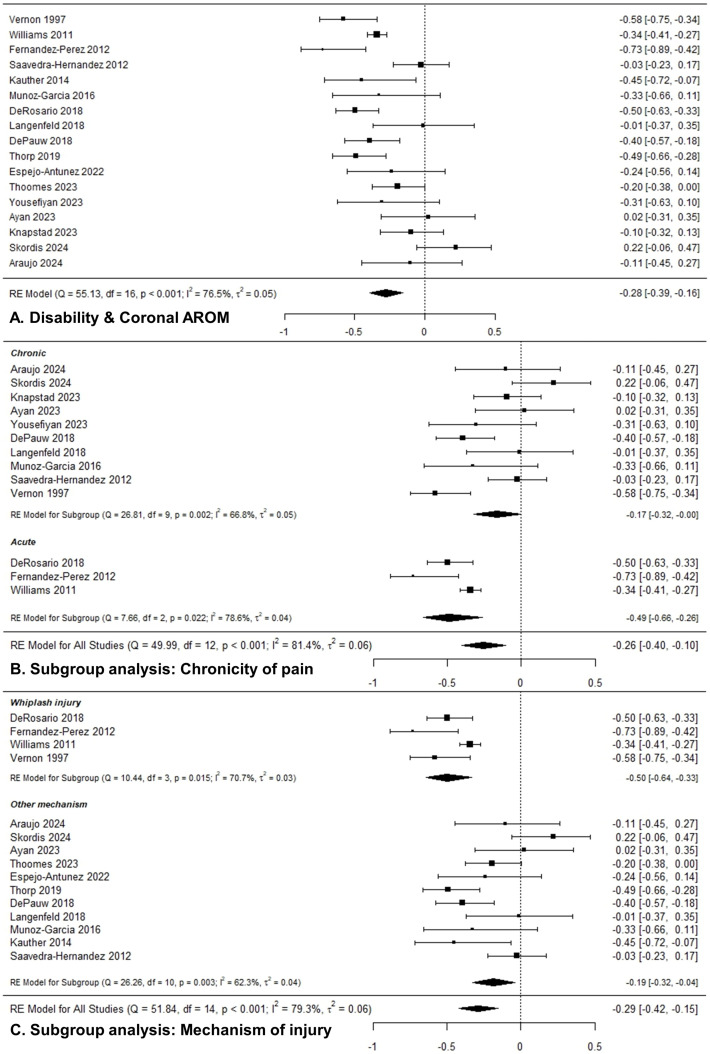
Overall and Subgroup Forest plots: Disability and Coronal AROM. (A) Overall random-effects meta-analysis (pooled Pearson’s r). (B) Subgroup analysis by chronicity of pain. (C) Subgroup analysis by mechanism of injury.

**Fig 9:**
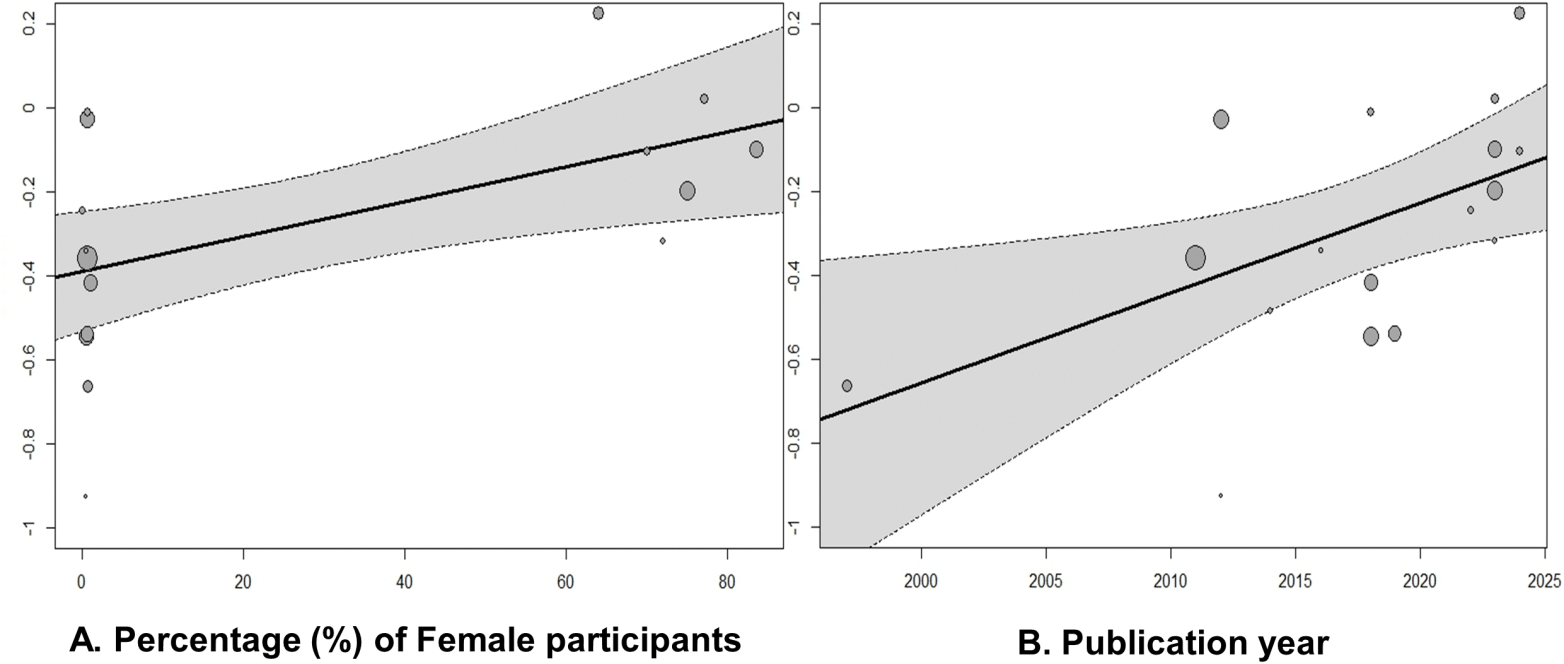
Meta-Regression Bubble Plots: Moderators vs Correlation (Disability & Coronal AROM). (A) Percentage of female participants vs Fisher’s z; (B) Publication year vs Fisher’s z.

Coronal AROM (17 studies, N = 1411) indicated a significant small-to-medium negative association (r = −0.28; 95%CI: −0.40 to −0.16; Fig 8A). Heterogeneity was substantial (I² = 76.6%). Meta-regression showed associations weakened with more female participants (Fig 9A, p = 0.01; R² = 39.5%) and recent studies (Fig 9B, p = 0.01; R² = 30.9%). Subgroups analyses showed stronger effects for acute/subacute (Fig 8B, p = 0.03; R^2^ = 29.8%) and WAD cases (Fig 8C, p = 0.007; R^2^ = 39.4%). No publication bias was found (Funnel plot, S4 Fig; Egger’s test, p = 0.63; Kendall’s τ, p = 0.36).

### Correlation between pain and AROM

a) Total AROM

**Fig 10:**
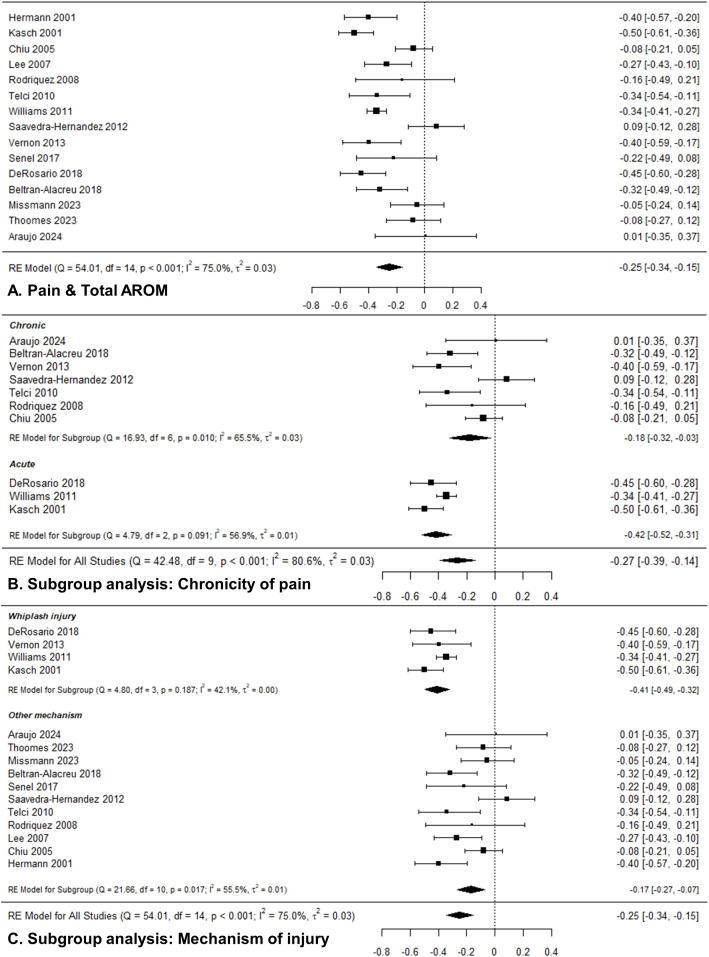
Overall and Subgroup Forest plots: Pain and Total AROM. (A) Overall random-effects meta-analysis (pooled Pearson’s r). (B) Subgroup analysis by chronicity of pain. (C) Subgroup analysis by mechanism of injury.

Total AROM (15 studies, N = 1888) had a significant small negative correlation (r = −0.26; 95%CI: −0.34 to −0.16; Fig 10A). Substantial heterogeneity was observed (I² = 74.9%). Subgroup analysis showed stronger effects in acute/subacute (Fig 10B; p = 0.01; R² = 48.2%) and WAD group (Fig 10C; p = 0.0009; R² = 59.0%). No significant moderators or publication bias found (Funnel plot, S5 Fig; Egger’s test, p = 0.4; Kendall’s τ, p = 0.7).

b) Sagittal Plane AROM

**Fig 11:**
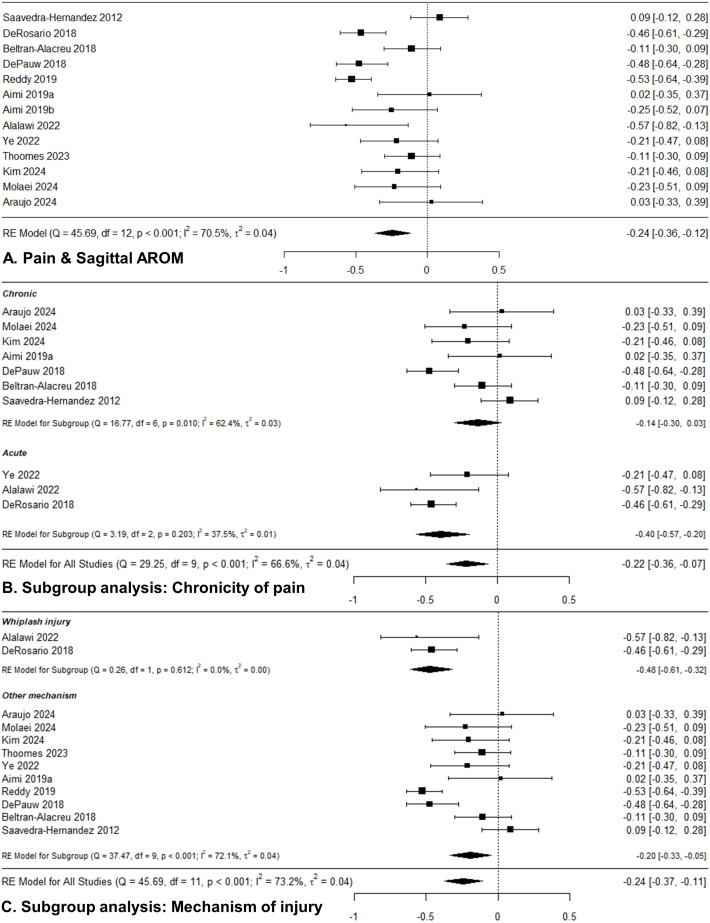
Overall and Subgroup Forest plots: Pain and Sagittal AROM. (A) Overall random-effects meta-analysis (pooled Pearson’s r). (B) Subgroup analysis by chronicity of pain. (C) Subgroup analysis by mechanism of injury.

Sagittal AROM (13 studies, N = 848) showed a significant small negative correlation (r = −0.24; 95%CI: −0.36 to −0.12; Fig 11A), and substantial heterogeneity (I² = 70.5%). Subgroup analysis showed stronger associations for acute/subacute (Fig 11B; R² = 26%) and whiplash group (Fig 11C; R² = 15.6%), though not statistically significant (p ≥ 0.7). No publication bias detected (Funnel plot, S6 Fig; Egger’s test, p = 0.6; Kendall’s τ, p = 0.9).

c) Transverse Plane AROM

**Fig 12:**
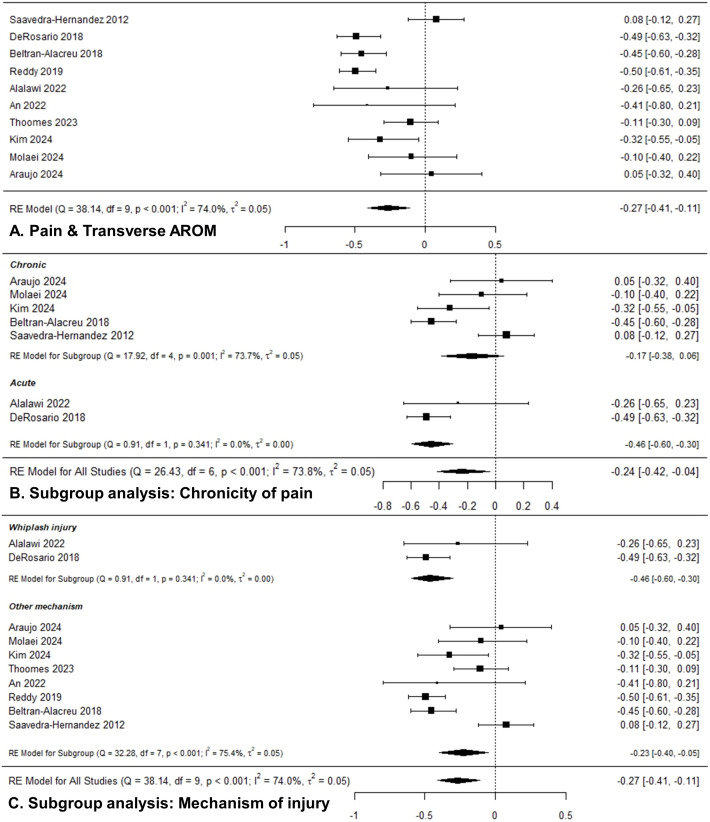
Overall and Subgroup Forest plots: Pain and Transverse AROM. (A) Overall random-effects meta-analysis (pooled Pearson’s r). (B) Subgroup analysis by chronicity of pain. (C) Subgroup analysis by mechanism of injury.

Transverse AROM (10 studies, N = 670) showed a significant small negative correlation (r = −0.27; 95%CI: −0.41 to −0.11; Fig 12A), with non-significant moderation or subgroups. Heterogeneity was substantial (I² = 74.04%). No publication bias was detected (Funnel plot, S7 Fig; Egger’s test, p = 0.5; Kendall’s τ, p = 0.7).

d) Coronal Plane AROM

**Fig 13:**
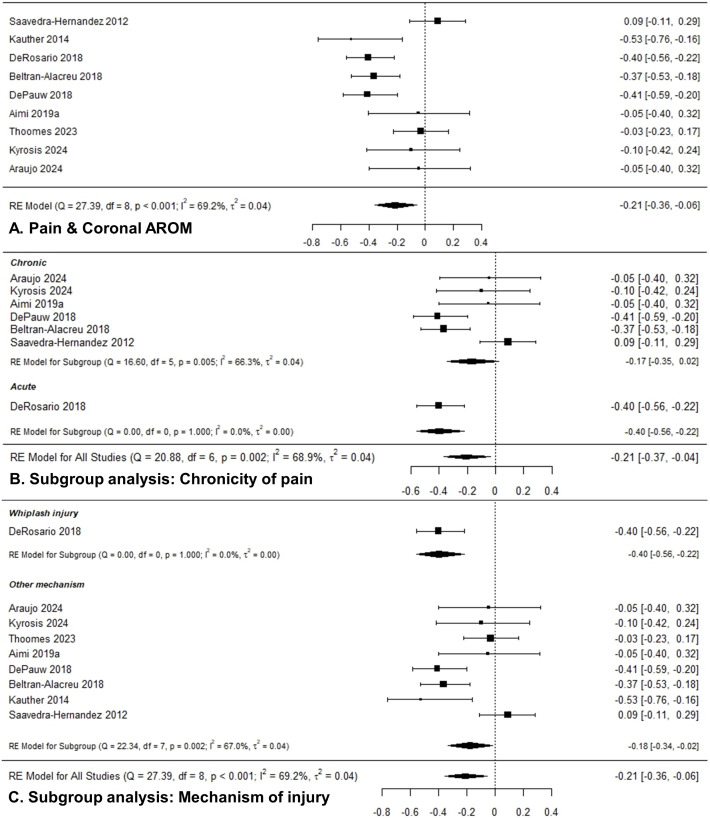
Overall and Subgroup Forest plots: Pain and Coronal AROM. (A) Overall random-effects meta-analysis (pooled Pearson’s r). (B) Subgroup analysis by chronicity of pain. (C) Subgroup analysis by mechanism of injury.

Coronal AROM (9 studies, N = 582) had a small negative correlation (r = −0.22; 95%CI: −0.36 to −0.06; Fig 13A), with no significant moderators or subgroups. Substantial heterogeneity was observed (I² = 69.18%). No publication bias was found (Funnel plot, S8 Fig; Egger’s test, p = 0.9; Kendall’s τ, p = 0.8).

### Comparison of correlations between pain and disability across planes of motion

Pairwise comparisons revealed no statistically significant differences between the pain and disability correlations across all planes (all p > 0.22, Table 3) despite disability correlation coefficients being slightly stronger. Similarly, no significant differences were found between planes for pain (p > 0.5) or disability outcomes (p > 0.3).

**Table 3:** Pairwise Z-test Comparisons between and across planes of movement.

Domain			Z^a^	P^b^
Same Plane^c^	Disability	Pain		
	Total	Total	−0.7	0.5
	Sagittal	Sagittal	−1.3	0.2
	Transverse	Transverse	−0.2	0.8
	Coronal	Coronal	−0.7	0.5
Across Planes^d^	Plane 1	Plane 2		
Disability	Total	Sagittal	0.7	0.5
	Total	Transverse	−0.2	0.8
	Total	Coronal	−0.3	0.8
	Sagittal	Transverse	−0.9	0.3
	Sagittal	Coronal	−0.9	0.4
	Transverse	Coronal	−0.1	0.9
Pain	Total	Sagittal	−0.1	0.9
	Total	Transverse	0.2	0.9
	Total	Coronal	−0.4	0.7
	Sagittal	Transverse	0.2	0.8
	Sagittal	Coronal	−0.3	0.8
	Transverse	Coronal	−0.5	0.6

^a^Z (difference test) compares two pooled Fisher’s z estimates. Negative values indicate a more negative pooled correlation for the first group/plane listed.

^b^Two-tailed p-values test the null of no difference.

^c^Same Plane rows compare Disability vs Pain within the same plane (Plane1 = Plane2).

^d^Across Planes rows compare Disability vs Disability and Pain vs Pain between planes.

## Discussion

This systematic review and meta-analysis quantified the cross-sectional association (i.e. Pearson’s *r*) between cervical AROM and patient reported measures of pain intensity or disability in people with neuromusculoskeletal NP. Overall, small to medium inverse correlations were found, indicating that reduced cervical AROM is modestly associated with higher levels of pain and disability. Disability showed slightly stronger associations (r ≈ −0.34 to −0.28) and narrower confidence intervals than pain (r ≈ −0.27 to −0.21), though the differences were not significant. Taken together, these findings suggest that cervical AROM reflects only part of the variance in patient‑reported pain and disability and should be interpreted as one component of a broader multidimensional assessment. Our results extend and statistically refine earlier narrative and systematic reviews that examined cervical AROM in NP populations by statistically pooling correlation coefficients.

Howell’s Narrative review highlighted that the relationship between NP, the NDI, and Cervical AROM may require further research [106]. Consistent with that narrative synthesis, our pooled estimates confirm that the association between AROM and disability lies in the small‑to‑medium range, while also narrowing the plausible range of effects and quantifying between‑study heterogeneity. Additionally, a review by Stenneberg et al. [12] showed that cervical AROM differs between people with NP, people with WAD, and asymptomatic controls; while Snodgrass et al. [8] summarized the broader clinical utility of cervical ROM for diagnosis, prognosis, and evaluating treatment effects. While assessment and interventions targeting AROM have been consistent features of rehabilitation for NP for decades [10,86] until now the magnitude of association between these two clinical constructs has been difficult to determine. By pooling correlation coefficients and exploring potential moderators this work contributes to better understanding of the nuanced connections between observable neck motion and patient reports of pain or disability.

Findings reveal small to medium inverse correlations, suggesting that reduced cervical AROM is modestly associated with higher levels of pain and disability. Specifically, disability showed slightly stronger associations (−0.34 ≥ r ≥ −0.28) and smaller confidence intervals compared to pain (−0.27 ≥ r ≥ −0.21). Such disparity is worthy of further consideration even though pairwise statistical comparisons did not confirm a significant difference (p ≥ 0.2). Stronger correlations between AROM and disability align with the function-focused items on most disability PROMs (e.g., driving, lifting) that are likely less affected by inter- or intra-day fluctuations than is pain severity, or perhaps highlighting differences in how pain and disability measures are scaled and calibrated by users. Though, the wider confidence intervals of AROM correlations with pain could also stem from greater daily pain fluctuations, or the effect of confounders such as psychological or social determinants of pain (e.g., psychological state on the test day [107]). It is important to note that correlations involving pain intensity at rest were extracted, as pain intensity at rest might be different from pain intensity during activity [108]. However, the context of pain assessment varied across included studies; for example some measured pain at rest during the test day [74], while some measured average pain experienced over the past week [66].

Our results also enable exploration of the relationship between PROs and AROM across different planes of motion. Although the sagittal plane showed a numerically stronger correlation with disability (r = −0.34; 95% CI: −0.43 to −0.25), statistical comparisons did not support differences across planes (p ≥ 0.3). Similarly, the strength of the association between AROM and pain is not statistically different across the sagittal, transverse, and coronal planes (p ≥ 0.6). Given the nature of study designs in this analysis (observational studies with cross-sectional analysis) causation cannot be inferred. However, interventions targeting restricted cervical AROM may lead to moderate improvements in both pain and disability, with greater changes expected in disability. As findings suggest, different effect sizes should be anticipated for pain and disability outcomes.

### Heterogeneity

Subgroup and moderator analyses revealed that substantial heterogeneity across studies was partly explained by differences in NP type and symptom duration, corroborating previous evidence and clinical guidelines [10,12]. Notably, studies primarily including participants with post-traumatic NP (e.g., whiplash [21,75,79,84,103]) reported stronger associations between AROM and the pain/disability PROMs compared to those with mixed or non-specific NP types. This pattern is compatible with findings from Stenneberg et al. [12], who observed reduced cervical AROM in patients with WAD compared to those with non-traumatic NP, and suggests that in post‑traumatic presentations, mobility impairments may be more tightly coupled with perceived disability. Duration of symptoms (chronicity) also moderated the association in some analyses, in which acute pain [75,84,103] showed stronger (i.e., more negative) associations between pain/disability and AROM than chronic conditions, possibly reflecting a greater contribution of mechanical impairment early in the course of symptoms and a more prominent role of psychosocial and central sensitization processes as pain becomes chronic [109]. However, not all prior work has identified robust differences in AROM between acute and chronic NP, and the confidence intervals around our moderator effects were wide, warranting cautious interpretation [12].

While some statistically significant moderators of effect were found, significant residual heterogeneity persisted after each meta-regression or subgroup analyses, such as those for Total AROM and disability. Although this variability might not change the clinical conclusions drawn from the evidence [57], it likely reflects the methodological differences across studies, including diverse outcome measurement tools (PROMs and AROM measures). As shown in S1 Table, studies assessed AROM using tools like goniometers (mainly CROM) [21,22,63,64,66–68,70,72,73,75,78–82,86,102–104], tape measure [71,85], and Multi-Cervical Unit [69,83]. These differing methods may have contributed to the observed heterogeneity. It is also important to note that while popular tools like CROM are widely used for cost-effectiveness and reliability [110,111], their limitations in assessing three-dimensional movements may have introduced additional confounders [36]. Additionally, diverse characteristics of study samples [71,82,83], lack of separate analyses for different groups of NP recruited in the study [74,78], inconsistencies in the reporting of inclusion and exclusion criteria [21,22,84,104], as well as variations in the specific occupations of participants [64,67,85], may have contributed to additional variability. As shown in Table 2 (and S3 Table), the GRADE assessment highlighted very low overall certainty of evidence, primarily due to high RoB from insufficient methodological reporting and small sample sizes. Nevertheless, mediation analysis of RoB scores across the eight meta-analyses did not reach significance and could not account for the variability in results, indicating no clear trends in reporting differing correlations among studies with high or low RoB.

### Strengths and limitations

This Systematic review and meta-analysis is the first to synthesize the association between AROM and PROs of pain and disability while examining NP types and patient characteristics as moderators. By pooling data across a broad range of clinical and research settings and using prespecified analytic strategies, we provide a more precise estimate of the strength of association between AROM and PROMs than has previously been available. A comprehensive search strategy (S1 File) was employed to minimize missed studies, increasing the diversity of patient populations, NP types, and measurement methods; but also contributing to heterogeneity and complicating synthesis. Studies that grouped all NP types without distinction were excluded from secondary analyses, which may have introduced reporting bias. As shown in S2 Table, many included studies were at high RoB (e.g., lack of outcome assessor blinding, small sample sizes), potentially skewing effect size estimates and reducing precision. Despite providing statistically significant findings and valuable insights, limitations related to study quality, inconsistency, and very low certainty of evidence warrant cautious interpretation.

### Implications for practice

The moderate correlation found in this review suggests that relying solely on PROMs or AROM may overlook important aspects of the patient’s condition. While it is commonly assumed in clinical practice that patients with MSK NP will exhibit reduced AROM, our findings indicate that these associations may be more nuanced. Notably, the relationship between AROM and pain or disability appears somewhat stronger in cases of acute NP and whiplash, but less so in other types of NP. These findings highlight the importance of individualized clinical assessment and the need to avoid overgeneralizing based on AROM alone.

The substantial heterogeneity observed in the meta-analyses might suggest underlying inconsistencies across studies, potentially including differences in how cervical AROM was measured. While our review did not directly assess measurement methods, the heterogeneity of pooled correlation coefficients could partly reflect the use of diverse assessment protocols and outcome measures. This possibility points to a need for more standardized approaches to measuring cervical AROM. Establishing and adopting consistent measurement protocols might help reduce variability, improve the reliability of clinical assessments, and enhance comparability across studies.

### Recommendations for future research

Future studies should explore the relationship between cervical AROM and disability in greater detail, particularly in populations with acute NP and WAD, where associations appeared stronger. Longitudinal research examining how changes in AROM correspond with fluctuations in pain and disability over time could offer valuable insight in the use of AROM as a clinical assessment tool.

Although the certainty of evidence regarding the cross-sectional association between AROM and PROMs remains very low, associations were generally consistent and negative. Nevertheless, high-quality research is needed to evaluate the potential of AROM as an indicator of individual progress in the long term.

In addition, the lack of standardization in AROM measurement methods across studies may have contributed to the observed heterogeneity. Developing and adopting standardized measurement protocols could enhance the consistency of future research findings.

## Conclusion

This review found a statistically significant small to moderate negative correlation between cervical AROM and self-reported pain intensity or disability, suggesting that decreased AROM is often associated with higher symptom severity. The association appeared somewhat stronger in cases of acute NP and whiplash, but less consistent in other types of NP. However, given the very low overall certainty of evidence (due to high RoB), and substantial heterogeneity, these findings should be interpreted with caution. While cervical AROM remains a useful component of clinical assessment, its interpretation should consider the broader clinical context. Future research should focus on high-quality studies that distinguish between NP types and evaluate the impact of rehabilitation on AROM and patient outcomes to build a stronger evidence base.

## Supporting information

S1 Fig Funnel plot: Disability and Total AROM. (JPEG)

S2 Fig Funnel plot: Disability and Sagittal AROM. (JPEG)

S3 Fig Funnel plot: Disability and Transverse AROM. (JPEG)

S4 Fig Funnel plot: Disability and Coronal AROM. (JPEG)

S5 Fig Funnel plot: Pain and Total AROM. (JPEG)

S6 Fig Funnel plot: Pain and Sagittal AROM. (JPEG)

S7 Fig Funnel plot: Pain and Transverse AROM. (JPEG)

S8 Fig Funnel plot: Pain and Coronal AROM. (JPEG)

S1 Table Characteristics of Included Studies. (XLSX)

S2 Table Details of Methodological Assessment (Risk of Bias) of Included Studies. (XLSX)

S3 Table Details of GRADE Evidence Profile. (XLSX)

S1 File MEDLINE Ovid search strategy. (DOCX)

S2 File The adapted Risk of Bias tool and explanations. (DOCX)

S3 File Excluded studies with specific reasons. (XLSX)

S4 File PRISMA 2020 checklist. (DOCX)
