# Supplementary figures and images for: Association between cervical spine clinical active range of motion and pain or disability in people with neuromusculoskeletal neck pain: A systematic review and meta-analysis
Source: PLoS One. 2026 Jul 24;21(7):e0353504. doi: 10.1371/journal.pone.0353504 (PMC13399312; doi:10.1371/journal.pone.0353504)

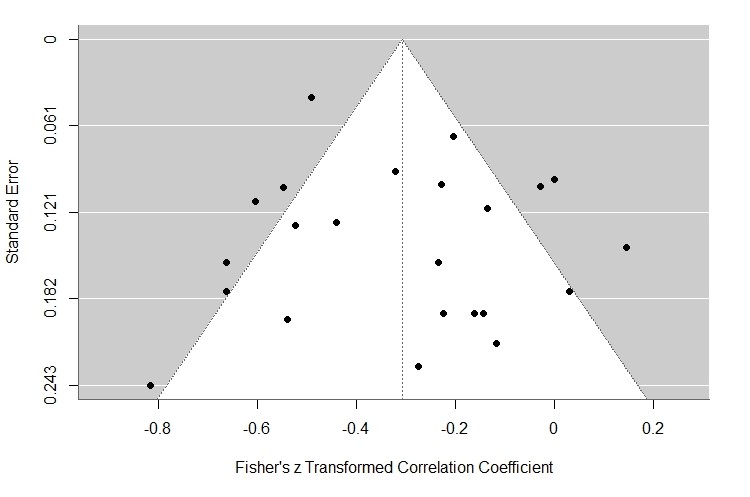

Supplement: S1 Fig — (JPEG) [file pone.0353504.s001.jpeg]

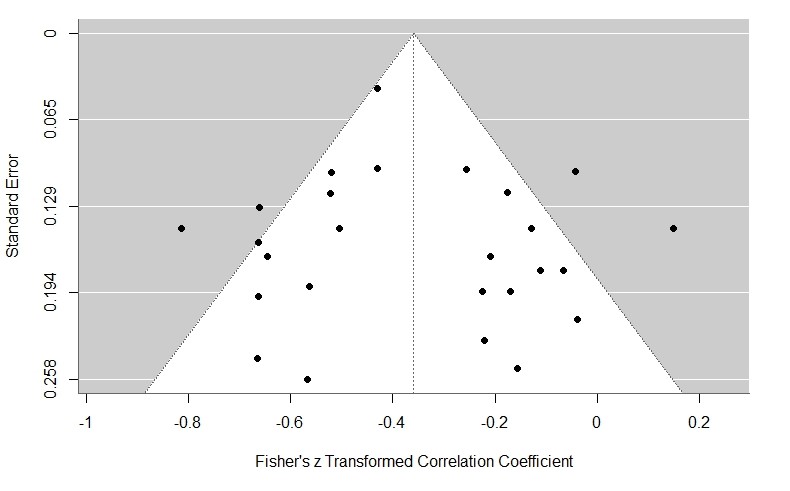

Supplement: S2 Fig — (JPEG) [file pone.0353504.s002.jpeg]

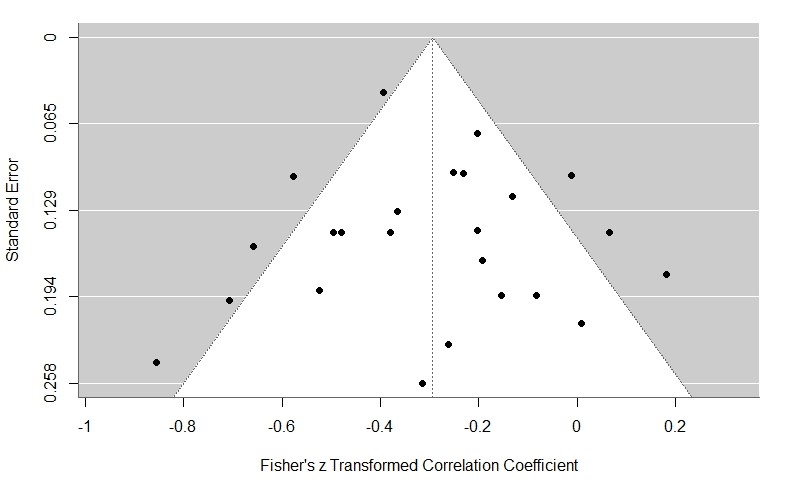

Supplement: S3 Fig — (JPEG) [file pone.0353504.s003.jpeg]

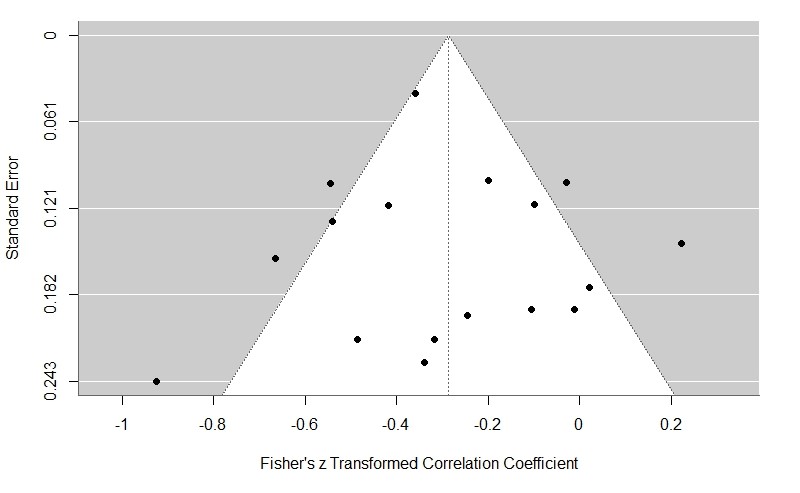

Supplement: S4 Fig — (JPEG) [file pone.0353504.s004.jpeg]

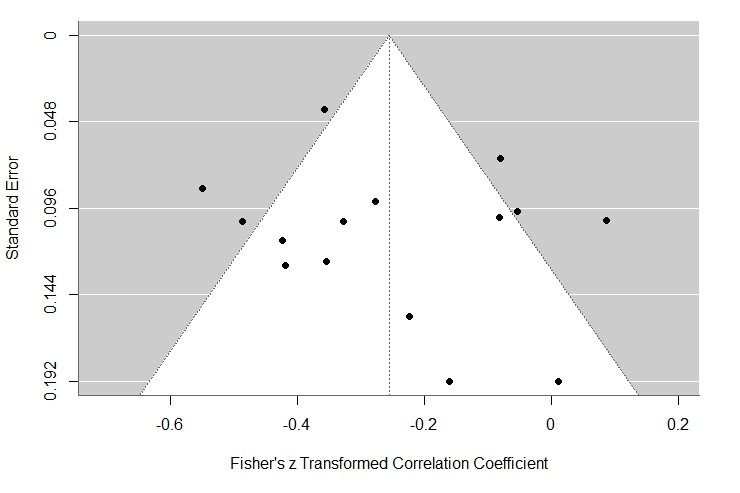

Supplement: S5 Fig — (JPEG) [file pone.0353504.s005.jpeg]

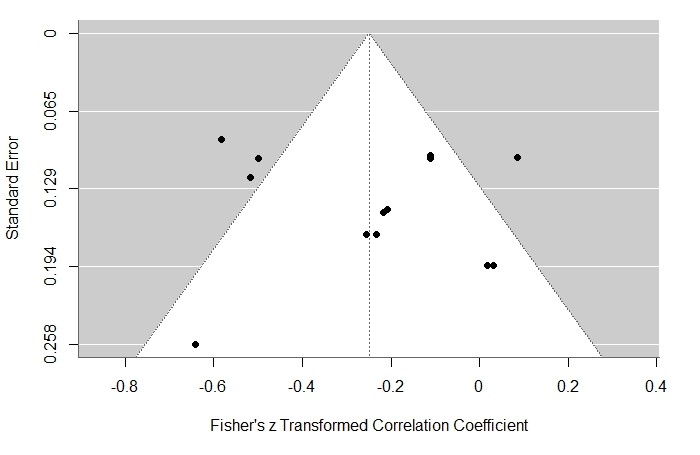

Supplement: S6 Fig — (JPEG) [file pone.0353504.s006.jpeg]

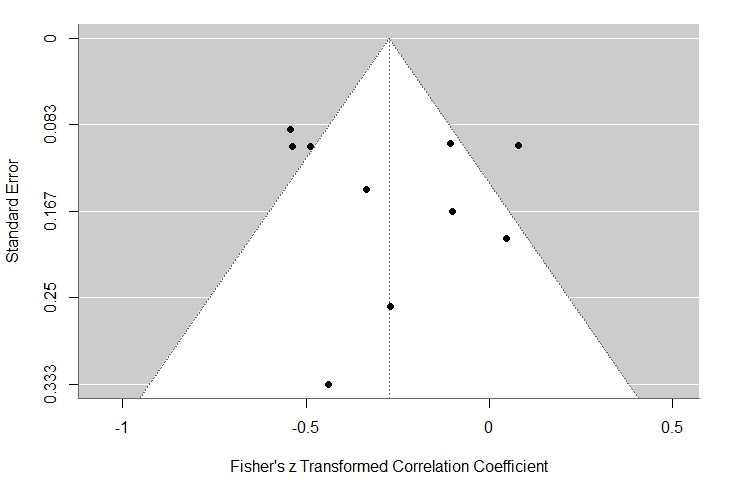

Supplement: S7 Fig — (JPEG) [file pone.0353504.s007.jpeg]

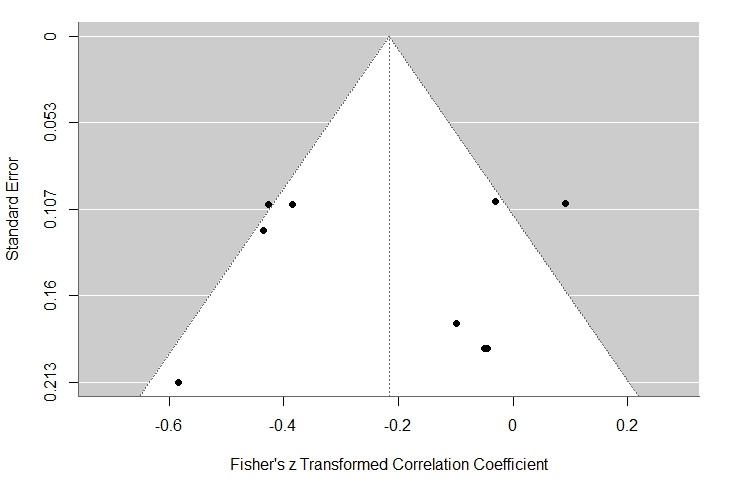

Supplement: S8 Fig — (JPEG) [file pone.0353504.s008.jpeg]
