# Supplementary material for: Association between cervical spine clinical active range of motion and pain or disability in people with neuromusculoskeletal neck pain: A systematic review and meta-analysis
Source: PLoS One. 2026 Jul 24;21(7):e0353504. doi: 10.1371/journal.pone.0353504 (PMC13399312; doi:10.1371/journal.pone.0353504)
Supplement: S1 File — (DOCX) [file pone.0353504.s012.docx]

The following search strategy was designed to systematically search existing evidence on the association between clinical biomechanical metrics of neck function (e.g., ROM, strength, acceleration, accuracy, smoothness, etc.) and subjective reports of pain and disability amongst adults with neuromusculoskeletal NP.

Given the volume of literature retrieved by this search, the first review focused on synthesizing the relationship between cervical AROM and PROMs.

# Ovid MEDLINE(R) Search Strategy

1 Neck Pain/

2 neck injuries/ or whiplash injuries/ or ((neck or cervic*) adj4 injur*).tw,kf.

3 ((neck or cervic*) adj4 (pain* or ache* or disabil*)).tw,kf.

4 (cervicalgia or Cervicogenic or cervicocephalic).tw,kf.

5 ((Radicul* or radiat*) adj5 (neck or cervic*)).tw,kf.

6 whiplash.tw,kf.

7 Radiculopathy/

8 Temporomandibular Joint Disorders/ or (temporomandibular adj4 (disorder* or disease* or dysfunction* or syndrome*)).tw,kf.

9 Biomechanical Phenomena/

10 Kinesthesis/

11 "Range of Motion, Articular"/

12 Movement/

13 Movement Disorders/

14 Proprioception/

15 ((biomechanic* or dynamic* or kinematic*) adj5 (head or neck or cervical)).tw,kf.

16 ((movement* or motion* or mobility* or motor*) adj5 (head or neck or cervical)).tw,kf.

17 ((position* or accuracy or velocity or accelerat* or jerk* or smooth*) adj5 (head or neck or cervical)).tw,kf.

18 (function adj5 (head or neck or cervical)).tw,kf.

19 ((strength or endurance) adj9 (head or neck or cervical)).tw,kf.

20 propriocept*.tw,kf.

21 (Sensorimotor or sensori-motor).tw,kf.

22 kinesth*.tw,kf.

23 1 or 2 or 3 or 4 or 5 or 6 or 7 or 8

24 9 or 10 or 11 or 12 or 13 or 14 or 15 or 16 or 17 or 18 or 19 or 20 or 21 or 22

25 23 and 24
