# Supplementary material for: Association between cervical spine clinical active range of motion and pain or disability in people with neuromusculoskeletal neck pain: A systematic review and meta-analysis
Source: PLoS One. 2026 Jul 24;21(7):e0353504. doi: 10.1371/journal.pone.0353504 (PMC13399312; doi:10.1371/journal.pone.0353504)
Supplement: S2 File — (DOCX) [file pone.0353504.s013.docx]

**Adapted form of the Quality Assessment Tool for Observational Cohort and Cross-Sectional Studies by National Institute of Health (NIH)**

|  | **Original items** | **Specifications and adaptations exclusively for this review** | **Article section** |
| --- | --- | --- | --- |
| 1. | Was the research question or objective in this paper clearly stated? |  | Method |
| 2. | Was the study population clearly specified and defined? | Demographic information, including description of age by range OR by mean and SD if clearly stated that only adults were included, description of recruitment location and timeframe | Selection |
| 3. | Was the participation rate of eligible persons at least 50%? | Number of persons fitting the eligibility criteria and the rate of those consenting to participate | Selection |
| 4. | Were all the subjects selected or recruited from the same or similar populations (including the same time period)?  Were inclusion and exclusion criteria for being in the study prespecified and applied uniformly to all participants? | The two questions were **divided into separate items below** to better represent differences between the studies:   1. Were all the subjects selected or recruited from the same or similar populations (including the same time period)? Were there separate analysis for different groups (if any)? 2. Were inclusion and exclusion criteria for being in the study prespecified and applied uniformly to all participants? | Selection |
| 5. | Was a sample size justification, power description, or variance and effect estimates provided? | An a priori sample size calculation had to be provided for detecting strength and direction of association between desired outcome measures *^a^*. | Selection |
| ~~6.~~ | For the analyses in this paper, were the exposure(s) *^b^* of interest measured prior to the outcome(s) *^b^* being measured? | All included studies being cross sectional analyses, this item has been **omitted** | - |
| 7. | Was the timeframe sufficient so that one could reasonably expect to see an association between exposure and outcome if it existed? | Were measurements of exposure (ROM) obtained on the same day as the outcome across all study participants? | Method |
| ~~8.~~ | For exposures that can vary in amount or level, did the study examine different levels of the exposure as related to the outcome (e.g., categories of exposure, or exposure measured as continuous variable)? | In this review, only active maximum range of motion of the neck is being studied as exposure which cannot vary in amount or level. Therefore, this item has been **omitted.** | - |
| 9. | Were the exposure measures (independent variables) clearly defined, valid, reliable, and implemented consistently across all study participants? | Were the neck range of motion outcome measures clearly defined, valid, reliable, and implemented consistently across all study participants? | Method |
| ~~10.~~ | Was the exposure(s) assessed more than once over time? | All included studies being cross sectional analyses, this item has been **omitted** | - |
| 11. | Were the outcome measures (dependent variables) clearly defined, valid, reliable, and implemented consistently across all study participants? | Were the patient-reported pain and disability measures clearly defined, valid, reliable, and implemented consistently across all study participants? | Method |
| 12. | Were the outcome assessors blinded to the exposure status of participants? | Were the ROM assessor(s) blinded to the measurement of pain/disability in participants? | Statistics |
| ~~13.~~ | Was loss to follow-up after baseline 20% or less? | All included studies being cross sectional analyses, this item has been **omitted** | - |
| 14. | Were key potential confounding variables measured and adjusted statistically for their impact on the relationship between exposure(s) and outcome(s)? | Were all measurements controlled for age, sex and other possible confounding variables? | Statistics |

*^a^* Suresh KP, Chandrashekara S. Sample size estimation and power analysis for clinical research studies. Journal of human reproductive sciences. 2012 Jan 1;5(1):7-13.

*^b^* Several items refer to “exposure (independent variable)” and “outcome (dependent variable)”, as the tool was primarily designed for cohort studies. For the purpose of this review, the independent variable was defined as neck range of motion, dependent variable defined as patient-reported pain and disability variables.

Ultimately, the adapted tool included 11 items, modified from the original 14-item version to better align with the context of cross-sectional designs relevant to this review. Details of the adaptation process are provided in the table above. Following the approach described by Pais et al. (2020)^[[1]](#footnote-1)^, studies were rated as having low risk of bias if they received >80% “Yes” responses (scores of 9-11), moderate risk of bias for 60-80% (scores of 7-8), and high risk of bias for <60% (scores of 1-6). These ratings were used to inform the GRADE assessment of overall evidence certainty.

1. Pais R, Ruano L, P. Carvalho O, Barros H. Global cognitive impairment prevalence and incidence in community dwelling older adults—a systematic review. Geriatrics. 2020 Oct 27;5(4):84. [↑](#footnote-ref-1)
